# Age ranges in breast cancer screening: simulated scenarios and analysis of benefits and harms

**DOI:** 10.1093/eurpub/ckac131.297

**Published:** 2022-10-25

**Authors:** M Pinto-Carbó, M Vanaclocha-Espi, J Martín-Pozuelo, P Romeo-Cervera, M Hernández-García, J Ibañez, S Castán-Cameo, NT Van Ravesteyn, O Zurriaga, A Molina-Barceló

**Affiliations:** 1 Cancer and Public Health, Foundation for the Promotion of Health and Biomedical Research of Valencia Region, Valencia, Spain; 2 INCLIVA Health Research Institute, Valencia, Spain; 3 Health Promotion Prevention in Health Environment, General Directorate of Public Health and Addiction, Valencia, Spain; 4 Public Health, Erasmus MC, University Medical Center Rotterdam, Rotterdam, Netherlands; 5 CIBERESP, Madrid, Spain; 6 Preventive Medicine and Public Health, Valencian University, Valencia, Spain

## Abstract

**Background:**

The Valencia Region Breast Cancer Screening Programme (VR-BCSP) (Spain) invites women aged 45-69 for mammography every 2 years (y). The aim is to evaluate benefits and harms of 3 age range scenarios of the VR-BCSP according to different adherence rates.

**Methods:**

Long-term impact simulation study (2020-2050) of 3 age range screening scenarios (S) for women ≥40y of the VR in 2020 (n = 1487000): S1, 45-69y (current VR-BCSP scenario); S2, 50-69y (excluding 45-49y) and S3, 45-74y (including 70-74y). A biennial screening interval was considered. The simulations were performed for 4 participation rates: A=current adherence (72.7%), B = +5%, C = +10% and D = +20%. Benefit indicators were: nº of BC in situ and invasive (screened vs. clinically detected), nº of BC deaths and % of BC mortality reduction. Harms indicators were: nº of false positives (FP) and % of overdiagnosis. Screening scenarios were simulated using the EUTOPIA evaluation tool.

**Results:**

Considering the current adherence, a reduction of BC mortality was observed in all scenarios (S1A=30.6%, S2A=27.9%, S3A=32.2%). In S2A the harms decreased vs. S1A: nº of FP (236vs423 x1000) and overdiagnosis (4.9%vs5.0%), but also the benefits: BC mortality reduction (27.9%vs30.6%) and nº of invasive BC screen detected (15/28vs18/25). In S3A vs S1A, an increase of benefits was observed: BC mortality reduction (32.2%vs30.6%) and nº of in situ BC screen detected (5/2vs4/3). On the other hand the nº of FP increased (460vs423 x1000), but overdiagnosis decreased (4.8%vs5.0%). All the results with an increased adherence had similar trend as the previous scenarios, showing a gradual increment in BC mortality reduction. Nevertheless overdiagnosis increase significantly in S3 (5.8% in all adherence increments), being higher than S1 (S1B=5.0%, S1C=4.9%, S1D=5.0%) and S2 (S2B=4.9%, S2C=4.9%, S2D=4.9%).

**Conclusions:**

The wider age range, the greater reduction in BC mortality but also the probability of FP and overdiagnosis.

**Key messages:**

• The wider age range, the greater reduction in BC mortality but also the probability of FP and overdiagnosis.

• This study provides a balance between benefits and harms of different screening scenarios allowing evidence-based decision making.

